# Factors Influencing the Phenotypic Characterization of the Oral Marker, PROP

**DOI:** 10.3390/nu9121275

**Published:** 2017-11-23

**Authors:** Beverly J. Tepper, Melania Melis, Yvonne Koelliker, Paolo Gasparini, Karen L. Ahijevych, Iole Tomassini Barbarossa

**Affiliations:** 1 Department of Food Science, School of Environmental and Biological Sciences, Rutgers University, New Brunswick, NJ 08901-8520, USA; btepper@sebs.rutgers.edu (B.J.T.); ivy.koelliker@gamil.com (Y.K.); 2 Department of Biomedical Sciences, University of Cagliari, Monserrato, Cagliari 09042, Italy; melaniamelis@unica.it; 3 Department of Reproductive and Developmental Sciences, IRCCS Burlo Garofolo, University of Trieste, Trieste 34137, Italy; paolo.gasparini@burlo.trieste.it; 4 College of Nursing, Ohio State University, Columbus, OH 43210, USA; ahijevych.1@osu.edu

**Keywords:** PROP tasting, psychophysical approach, age, gender, weight status

## Abstract

In the last several decades, the genetic ability to taste the bitter compound, 6-*n*-propyltiouracil (PROP) has attracted considerable attention as a model for understanding individual differences in taste perception, and as an oral marker for food preferences and eating behavior that ultimately impacts nutritional status and health. However, some studies do not support this role. This review describes common factors that can influence the characterization of this phenotype including: (1) changes in taste sensitivity with increasing age; (2) gender differences in taste perception; and (3) effects of smoking and obesity. We suggest that attention to these factors during PROP screening could strengthen the associations between this phenotype and a variety of health outcomes ranging from variation in body composition to oral health and cancer risk.

## 1. Introduction

Taste perception varies from person to person, strongly influencing food preferences and health [1]. In the last several decades, numerous studies have focused on the use of the bitter compound, 6-*n*-propylthiouracil (PROP) and its chemical relative phenylthiocarbamide (PTC), as genetic markers for oral sensations that have downstream effects on food preferences, eating habits, nutritional status and health [1,2,3]. This approach is based on data indicating that PROP taster status is associated with variations in taste perception and preference for a wide range of oral stimuli including other bitter substances [4,5,6,7,8,9], chemical irritants [10,11], sweet substances [12], sour compounds [13], umami taste [14], fats and high-energy foods [15,16,17], compounds which give rise to astringent sensations [18], and fruits and vegetables [19,20,21]. Some studies suggested that PROP-related sensory differences may be extended to the olfactory system [22,23], and that PROP taster status may influence food perception also via aromas or flavors [24,25]. Relationships between PROP taster status and health indicators such as body mass index (BMI) [26,27], antioxidant status [28], colonic neoplasm risk [29,30,31] and respiratory function [32] have been widely reported.

The validity of PROP tasting as a biomarker depends on the use of robust PROP phenotyping methods. Common screening procedures are based on psychophysical approaches which fall into two general classes: threshold and suprathreshold methods. Threshold measures determine the lowest PROP concentration (or amount) that can be distinguished by an individual. These measures are reliable with a long history of use in the field. They can effectively separate individuals who detect PROP only at high concentrations or not at all (non-tasters), from those who perceive PROP as bitter (tasters). However, thresholds do not distinguish individuals who perceive PROP as extremely bitter (super-tasters) from those who perceive it as moderately bitter (medium tasters) [1,33].

Suprathreshold methods utilize rating scales to assess PROP bitterness across the psychophysical range. Supratheshold scaling can distinguish individuals who are super-tasters from medium tasters, thus classifying individuals as belonging to one of the three PROP taster categories [1,34,35,36,37,38]. Suprathreshold methods are based on ratings of the perceived intensity of PROP following stimulation with multiple samples, a single solution or impregnated filter papers [34,35,36,39]. 

Bartoshuk and colleagues [34] first pioneered PROP screening methodologies linking PROP intensity ratings with a classification scheme. In the original method, five different concentrations of PROP and five concentrations of sodium chloride were presented to assessors who were classified into groups based on the relative intensity of PROP to NaCl. Those who gave higher ratings to PROP than NaCl were classified as super-tasters. Those who gave similar ratings to both stimuli were classified as medium tasters and those who gave higher ratings to NaCl relative to PROP were classified as non-tasters. Some workers have called into question the validity of NaCl ratings as an independent stimulus [40,41]. This conclusion may be based, in part, on data showing that at high NaCl concentrations (>0.35 M), NaCl ratings are not independent of PROP taster status and diverge in super tasters [41]. Nevertheless, the use of NaCl as a reference standard has been successfully employed by numerous laboratories across the globe [17,18,27,33,34,38,39,42,43,44,45,46,47,48,49,50,51,52,53,54,55,56,57,58,59,60,61]. Data described later in this review illustrate this point.

The two predominant scales used in PROP screening are the labeled magnitude scale (LMS) [62], and its variant, the general labeled magnitude scale (gLMS) [63]. Both scales give subjects the freedom to rate the perceived intensity of PROP relative to the ‘strongest imaginable oral sensation’ they had ever experienced (LMS) or the ‘strongest imaginable sensation of any kind’ they had ever experienced, including those evoked by the strongest sound, pain, or light (gLMS). Training in how to use these scales is typically provided to participants in advance.

The need for brief screening methods that could be employed outside of a traditional laboratory setting prompted Tepper and colleagues [35,36] to develop a series of related methods based on the original technique of Bartoshuk et al. [34]. These methods include the 3-solution and 1-solution methods [35] and the paper disk method [36]. The 1-solution and paper disk methods rely on empirically-derived cutoff scores for PROP taster classification. These techniques have been used in numerous investigations in our work [17,18,27,33,38,39,43,45,46,47,48,49,50,51,52,53,54,55,56,57] and the work of others [58,59,60,61]. PROP classifications obtained with this suite of methods is highly correlated with all known *TAS2R38* genotypes [27,33,64,65,66,67] and differences in physiological responses such as prefrontal cortex activity [68] and degree of activation of peripheral taste function [69,70].

It has become our practice in recent years to define taster status primarily on the basis of PROP cutoff scores unless a participant gives a ‘borderline’ rating to PROP [18,39,52,54,55,56]. Only in rare instances (~4–6% of the time) is the NaCl rating needed to clarify an individual’s PROP classification. We continue to administer the NaCl disk to reliably classify this small proportion of individuals and to preserve the integrity of the method.

PTC tasting was first described by Fox [71] and was identified as a genetic trait with Mendelian-like inheritance almost 60 years ago [72]. Since then, it has become clear that PROP/PTC tasting is a complex trait, with many factors involved in its determination. First, alleles of the gene that code for the G-protein-linked receptor *TAS2R38* explain most of the phenotypic variation in PROP tasting [73,74]. The allelic diversity of this gene, located on chromosome 7 [75], is due to five single-nucleotide polymorphisms (SNPs) [76]. Three of these SNPs, located at base pairs 145 (C/G), 785 (C/T), and 886 (G/A), result in three amino acid substitutions (Pro49Ala, Ala262Val, and Val296Ile) which give rise to 5 distinct haplotypes (PAV, AVI, AAV, AAI, PAI, and PVI which is very rare) [74]. There are two common haplotypes, PAV the dominant (taster) variant and AVI the recessive (non-taster) one. Typically, non-tasters are homozygous for the AVI haplotype, whereas considerable genotypic overlap between the medium and super-taster groups has been reported [11,27,74]. Rare haplotypes (AAV, AAI, and PVI) have been shown to contribute to intermediate sensitivity [67] and are more frequent in African populations [76]. Also, differences in *TAS2R38* expression have been strongly associated with PROP bitterness intensity [77]. The *TAS2R38* receptor binds synthetic thiourea derivates, such as PROP and PTC, as well as natural ligands, such as glucosinolates of the *Brassica* family. All of these compounds contain the thiocyanate moiety (N–C=S) which is responsible for their bitter taste [71,78].

Studies also suggest that other modifying genes may be involved in PROP tasting [79,80,81]. For example, a polymorphism in the gene that codes for zinc binding capacity of the salivary protein, gustin (CAVI) [38], has been shown to affect PROP sensitivity by acting as growth factor for taste buds [65]. The discovery of this polymorphism in Gustin provided the first mechanistic explanation for why PROP super-tasters, compared to non-tasters, are more responsive to a wide range of stimuli that are not mediated via the *TAS2R38* bitter receptor. Numerous studies have reported that PROP super-tasters have higher papillae densities on the tongue tip than non-tasters [12,34,65,82,83,84]. However, subsequent work by Barbarossa et al. [39] clarified this relationship by showing that fungiform papilla density was more strongly related to variation in the gustin gene than to PROP tasting or *TAS2R38* polymorphisms. Together, these findings suggest that there is functional cooperation between these two genes products to influence orosensory perceptions, and these effects are mediated by taste papilla density. It is important to note, however, that other studies failed to find associations between PROP tasting and gustin genotypes [37,85]. Finally, greater PROP sensitivity has also been related to differences in the secretion of other salivary chemical components including proline-rich proteins and other classes of proteins involved in bitter taste and astringency perception [18,45,64,71,86].

Despite the accumulating evidence supporting associations between PROP tasting, nutrition and health, these relationships remain controversial due to the many null reports in the literature [87,88,89,90,91,92]. These inconsistencies may be due, in part, to underlying factors that modify the phenotypic expression of this trait. The purpose of this review is to highlight common factors identified in our research such as age, gender, obesity and smoking that can influence the characterization of the PROP phenotype. Accounting for these factors could lead to more precise phenotyping and more robust relationships between this biomarker, nutrition and health. All of the data presented in this review were collected in individuals under conditions of unobstructed air flow (without nose clips).

## 2. Gender and Age Effect

The effects of gender and age on chemosensory perceptions are well known [93,94], but they have been less well studied in relation to PROP sensitivity. Several studies reported a gender difference in PROP perception. Although this difference might be due to hormonal levels, these data showing that women were more sensitive to PROP bitterness than men, were more likely to be tasters [34,48,95], or super-tasters, [34] and had more taste buds and fungiform papillae [34,40]. However, other reports do not substantiate this difference [65,96,97].

Age has also been associated with moderately higher PROP thresholds, accounting for 5–8% of the taste acuity variance [37,65,95,98]. Mennella et al. showed that among *TAS2R38* heterozygotes (PAV/AVI), children were more responsive to PROP than adults [99]. These data suggest that the penetrance of this gene (the extent to which environmental factors such as age, influence the phenotypic expression of this trait), varies with age [99]. Further, children have more papillae on the tip of the tongue and they perceive greater intensity from the basic tastes than do adults [100]. These anatomical and functional characteristics could explain why age indirectly affects the PROP genotype-phenotype relationship.

**Figure 1:**
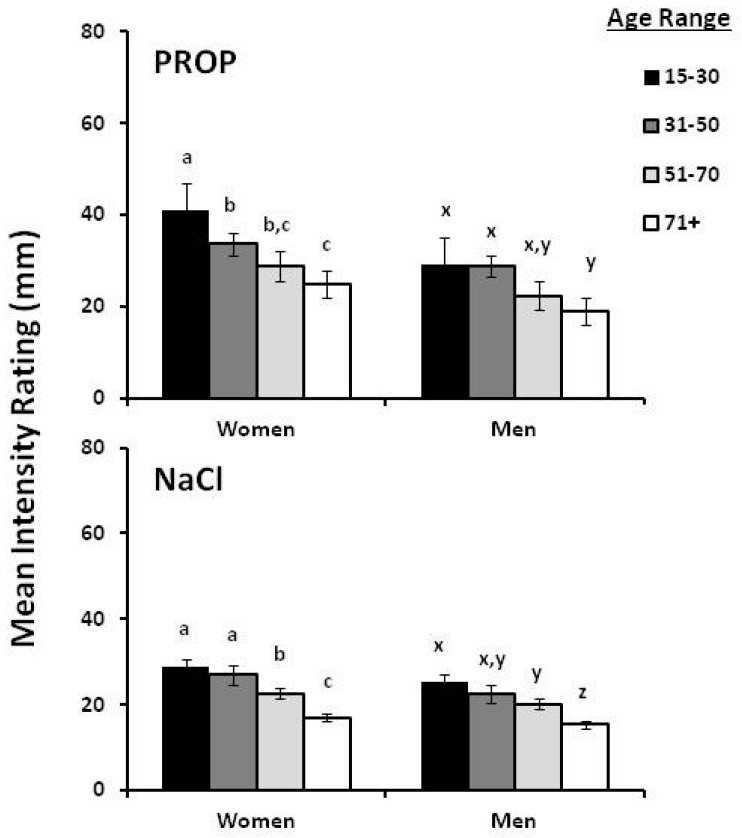
Mean (±Standard Error, SE) 6-*n*-propylthiouracil (PROP) (**top**) and NaCl (**bottom**) ratings across age groups in both women and men from the isolated community of Val Borbera, Northwest Italy. *n* = 589; sample size per age group: 59 (15–30 years); 191 (31–50 years); 261 (51–70 years); 78 (71+ years). Ratings by men and women were analyzed separately. For women, mean values within a stimulus type (PROP or NaCl), with different superscripts (a, b and c) are significantly different as a function of age; superscripts x, y and z are used to denote age differences for men. Significant differences, within a stimulus type (PROP or NaCl), are indicated by different letters (a, b and c are used to denote differences for women, while x, y and z for men). For all comparisons, *p* < 0.001 by Duncan’s Multiple Range test subsequent to ANOVA. Unpublished data from Tepper et al. [107].

It is well known that population heterogeneity is a common contributor to the problem of non-replication when complex traits, such as PROP phenotype, are under study [101]. Since ethnic homogeneity can reduce noise by diminishing ancestral diversity [101,102,103], we studied gender and age-related changes in the bitterness of PROP and NaCl in 589 adults (18–96 years of age; 343 females; 246 males) residing in the isolated community of Val Borbera located in northwest Italy. All participants were free-living and healthy by self-report, and none were taking medications that might interfere with taste function. Half of the participants were >50 years of age. Participants rated the intensity of PROP and NaCl—impregnated filter papers according to the method of Zhao et al. [36]. Among the youngest participants (15–30-year-old), women gave higher intensity ratings to PROP than men (*p* < 0.001). PROP ratings declined slowly and consistently with age in both genders (all *p*-values < 0.001) (Figure 1). These changes presumably reflect well-documented decreases in papillae density, shape, vascularization and function that occur with the aging process [104,105,106] and not a unique effect of *TAS2R38* gene on taste function. However, changes in expression of genes related to taste function cannot be excluded as a possibility for decreased PROP perception.

**Figure 2:**
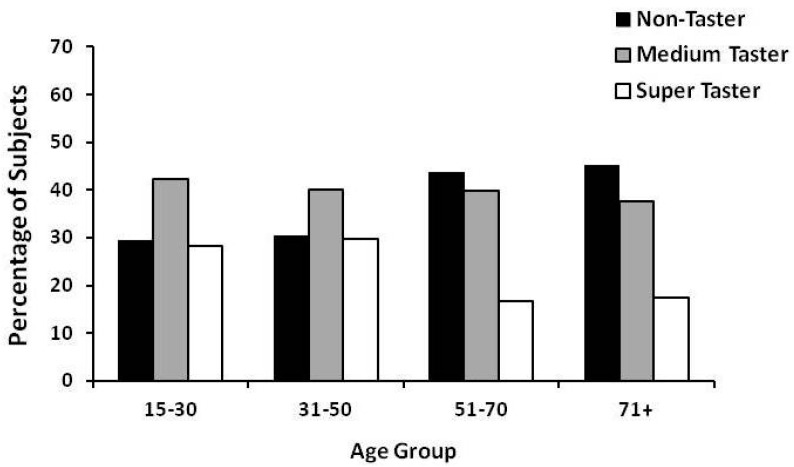
Distribution of PROP taster groups by age in the isolated community of Val Borbera, Northwest Italy. *n* = 589; sample size per age group: 59 (15–30 years); 191 (31–50 years); 261 (51–70 years); 78 (71+ years). The percentage of non-tasters increased and the percentage of super-tasters decreased in those >50 years of age (by Chi-square analysis; *p* < 0.001). Unpublished data from Tepper et al. [107].

When participants (both men and women) were classified by PROP status (Figure 2) the distribution of taster groups agreed with population norms for western European populations, up to the age of 50 years. After age 50, the percentage of non-tasters increased and the percentage of super-tasters declined by almost 50%. These data suggest that age-related taste loss could have implications for PROP screening and phenotyping, especially in geriatric populations.

## 3. Weight Status Effect

It has often been hypothesized that chemosensory perceptions are blunted in obesity. This supposition is based on evidence showing reductions in oral fat sensation [108,109,110], sweet taste [111], bitter and salty taste [112], umami taste [113] and general taste and smell ability [114] among populations with obesity. However, some studies show no apparent disruptions in the perception of fattiness and sweetness from food products in obese individuals [52], or in the oral detection of fatty acids [115]. Indeed, other studies have come to a different conclusion, showing greater acuity for sweet and salty taste in obese relative to lean individuals [116]. 

A major focus of our work is on understanding the role of PROP tasting as a biomarker for excess fat/energy intake and increased adiposity [1,3]. Two studies conducted exclusively in women, showed an inverse association between PROP tasting and BMI, i.e., non-taster women were heavier than super-taster women [46,48]. Another mixed-gender study reported this same negative association between PROP tasting and BMI that was specific to women but not men [27]. However, other studies failed to replicate these findings reporting no associations between PROP tasting and weight status in either gender [44,90,91,92,117,118,119,120,121]. 

**Figure 3:**
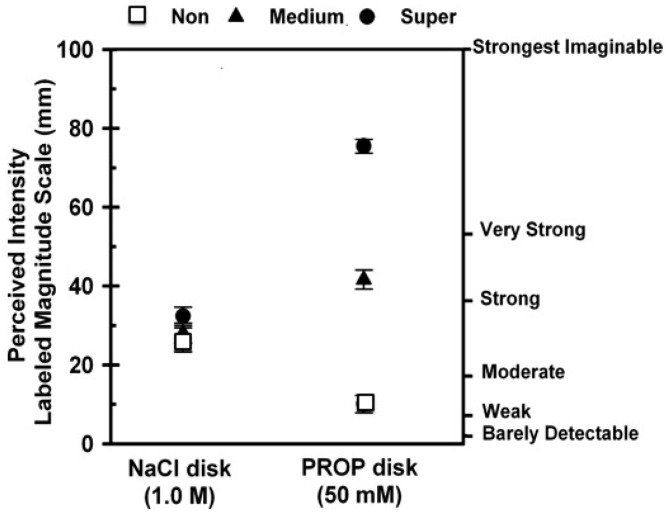
Classification of 93 lean women, 18–45 years of age as PROP non-tasters, medium tasters and super-tasters based on intensity ratings (mean ±95% confidence) for paper disks that were impregnated in PROP (50 mM) and NaCl (1.0 M), and then dried. The PROP disks contained 0.28 mg (±2.2% CV) PROP measured by ethanol extraction. Super-tasters were identified with a cutoff score for PROP bitterness >67 mm on the LMS; non-tasters had a cutoff score ≤15 mm. All others were identified as medium tasters. There were no differences in the perception of NaCl across the three subject groups. Data are from Tepper et al. [28] with permission.

Most studies examining associations between PROP tasting and adiposity have been conducted in mixed subject groups of overweight-obese individuals. Only a few recent studies have been conducted in individuals who meet the criterion for obesity (BMI > 30 kg/m^2^) [56,120,122]. Presumably, if taste function is altered in obesity, this could impact PROP phenotyping procedures. Two studies described below illustrate this effect. Figure 3 shows typical PROP and NaCl ratings obtained from a group of lean, young women classified by PROP status where NaCl ratings were centered at ~30 mm and did not vary across PROP taster groups. 

**Figure 4:**
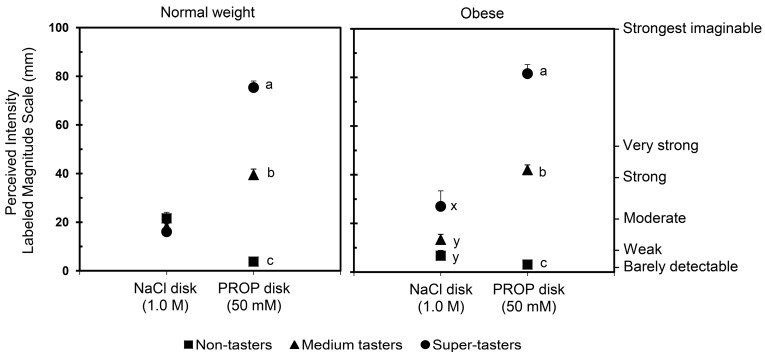
Mean (±SE) values of perceived intensity NaCl and PROP in an ethnically homogeneous cohort of Sardinia, Italy. *n* = 134 (48 Males, 86 Females) normal weight subjects; *n* = 98 (45 Males, 53 Females) obese subjects. Values with different superscripts (a, b, etc.) indicate significant differences (*p* < 0.034; Duncan’s test subsequent 2-way-ANOVA).

Figure 4 shows the PROP and NaCl ratings from a sample of normal weight (mean BMI = 21.85 ± 0.177 kg/m^2^) and obese men and women (mean BMI = 35.32 ± 0.61 kg/m^2^). Results showed that the NaCl ratings of non-tasters and medium tasters with obesity were lower than those of super-tasters with obesity (*p* = 0.0015 and *p* = 0.000094, respectively; Duncan’s test subsequent two-way ANOVA). No differences in NaCl ratings were observed across taster groups among normal weight individuals (*p* > 0.05). Why non-tasters and medium tasters with obesity seem to experience diminished taste responsiveness to NaCl is unknown, and deserves further study. These data suggest that investigators should use caution when interpreting NaCl ratings used as a reference standard in PROP screening among individuals with obesity. 

A second issue to consider is that the presence of obesity might alter the relationship between PROP tasting and body weight. Specifically, Carta et al. [120] found that super-tasters who were lean (BMI < 25 kg/m^2^) had a lower BMI than medium or non-tasters who were lean, consistent with previously reported findings among overweight individuals. However, this relationship was reversed among individuals with obesity (BMI > 30 kg/m^2^) such that super-tasters with obesity had a higher BMI than medium tasters and non-tasters with obesity. 

## 4. Smoking

Nicotine from cigarettes is both bitter tasting and pungent [123,124]. Aversion to the negative chemosensations of nicotine is thought to reduce the risk of smoking or the severity of nicotine dependence in those who do smoke [125,126]. Several studies have shown that those who are taste blind to PROP (or AVI homozygotes) may be at greater risk for smoking, since non-tasters may be less responsive to the aversive taste of nicotine [125,127,128]. Whether smokers can learn to like the taste of nicotine due to its stimulant properties, is an open question. 

**Figure 5:**
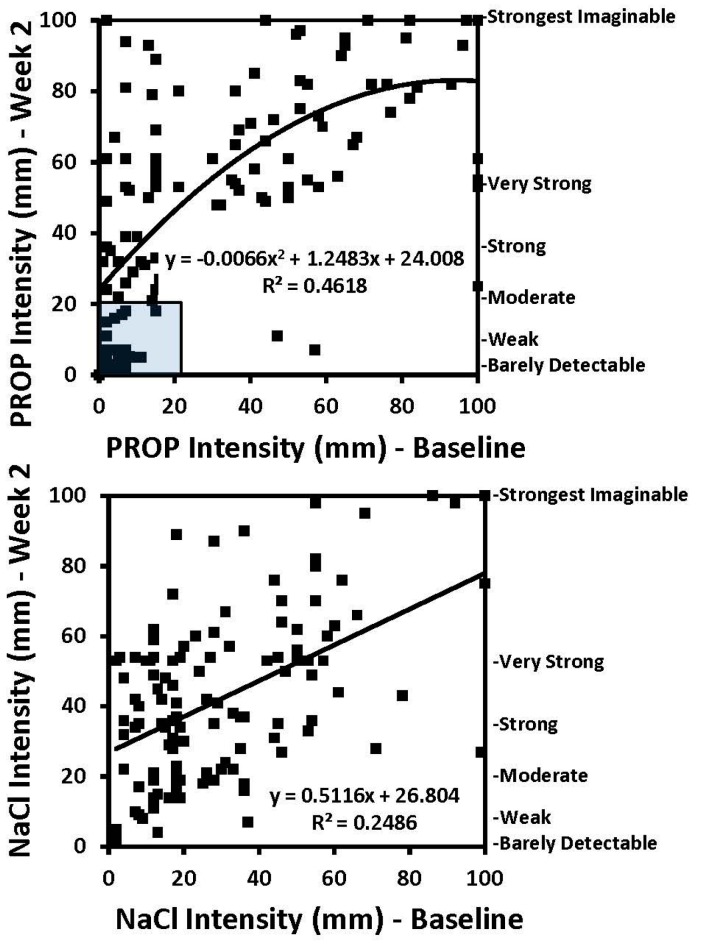
Regression of intensity ratings for 6-*n*-propylthiouracil (PROP; **top panel**), and NaCl (**bottom panel**), at baseline and at two weeks after smoking cessation (controlling for age and gender). Intensity ratings were made using the Labeled Magnitude Scale (0–100 mm). Intensity ratings for both stimuli increased across the trial. PROP intensity ratings have a logarithmic trend line, while NaCl ratings have a linear trend line. Mean intensity ratings for both stimuli rose from baseline to week 2 (for NaCl, 30.2 ± 2.1 vs. 42.3 ± 2.1 and for PROP, 35.9 ± 3.3 vs. 53.2 ± 2.9; *p* < 0.01 for both). *n* = 120. The shaded box identifies a small group of participants who were classified as PROP non-tasters at both. Data are from Ahijevych et al. [57] with permission.

Smoking is also known to diminish both taste and smell sensations, whereas smoking cessation (in the range of 3–6 months duration) partially or fully restores these functions [129,130,131]. Very little work has been done on the acute effects of smoking abstinence, although one study showed partial recovery of taste acuity following 12 h of smoking abstinence [132]. To our knowledge, the study by Ahijevych et al. [57] is the only clinical trial to examine relationships between PROP tasting and smoking cessation in individuals with access to nicotine replacement therapy (NRT). Results showed that PROP non-tasters were more likely to use nicotine lozenges, which is consistent with the notion that oral nicotine exposure is less aversive to non-taster individuals. As a consequence of conducting this trial, we screened 120 smokers (consisting of different races and both genders) for PROP status at baseline and again after two weeks of smoking abstinence (with access to NRT). We regressed the PROP ratings at baseline against the PROP ratings at two weeks to model the changes in PROP taste intensity following the abstinence period; the same analysis was conducted for the NaCl ratings. As shown in Figure 5, most participants gave higher intensity ratings to both PROP and NaCl at week 2 than they did at baseline. Indeed, 30% of participants shifted from PROP non-taster status to PROP taster status by the end of the trial. Only a small group of participants (identified in the shaded box; top panel) were identified as PROP non-tasters both at baseline and after two weeks of abstinence. These data demonstrate that smoking-associated taste loss can impact PROP phenotyping procedures, overestimating the true proportion of PROP non-tasters in smoking trials. Our findings also reveal a rapid recovery of suprathreshold taste intensity after just two weeks of smoking cessation. This finding could have broader implications for understanding the role of inhaled smoke on chemosensory insult and recovery of function.

## 5. Conclusions

The data reviewed here suggest that a variety of common characteristics including gender, age, obesity and smoking that are known to influence taste function can also affect PROP phenotyping. These individual effects may be compounded in study populations that include both genders, and participants of variable age, body weight and smoking status. Thus, researchers, clinicians and other practitioners should use caution when phenotyping diverse populations for PROP taster status, and pay close attention to the demographic characteristics of the individuals under study. Statistical procedures to adjust for the effects of these variables should be considered, when feasible. 

PROP tasting is a popular tool that has led to important insights into food preferences, eating behavior, obesity and metabolic diseases such as cancer. A possible link between this phenotype or *TAS2R38* gene and alcoholism has been claimed [11,133]. More recently, it has been used as a clinical assessment tool in a weight loss intervention [56] and as a biomarker for risk of dental caries [134,135] and upper respiratory infections [32]. Understanding the underlying characteristics of this phenotype is a critical first step for exploiting its utility as a biomarker in health and disease. As phenotypic variation in PROP sensitivity is genetic in origin, we suggest that PROP phenotype is a useful biomarker for nutrition, health and disease because it can be assessed in large populations with scientific rigor, at low cost and non-invasively.
